# Knowledge, counseling practices, and educational gaps related to drug–food interactions among healthcare professionals in Saudi Arabia: a cross-sectional study

**DOI:** 10.3389/fmed.2025.1719936

**Published:** 2025-12-16

**Authors:** Abdulaziz Shalali, Saad M. Wali, Mohammed M. Aldurdunji

**Affiliations:** 1 College of Pharmacy, Umm Al-Qura University, Makkah, Saudi Arabia; 2 Department of Pharmacology and Toxicology, College of Pharmacy, Umm Al-Qura University, Makkah, Saudi Arabia; 3 Department of Pharmaceutical Practices, College of Pharmacy, Umm Al-Qura University, Makkah, Saudi Arabia

**Keywords:** drug–food interactions, pharmacist counseling, cultural competency, pharmacovigilance, healthcare education

## Abstract

**Background:**

Drug–food interactions (DFIs) can significantly affect therapeutic efficacy and patient safety. However, there is limited evidence regarding healthcare professionals’ knowledge and practices concerning DFIs, particularly in Saudi Arabia. This study aimed to evaluate the awareness, counseling practices, and perceived barriers among healthcare professionals regarding DFIs, with a focus on cultural and educational dimensions.

**Methods:**

A cross-sectional survey was conducted among 385 healthcare professionals in Saudi Arabia using a structured self-administered questionnaire. The survey assessed knowledge of DFIs, sources of information, counseling behavior, reporting practices, and the perceived need for additional training. Descriptive statistics were used to summarize findings.

**Results:**

A total of 231 respondents (60.0%) demonstrated limited knowledge regarding DFI mechanisms. More than half (51.4%) reported receiving no formal instruction on DFIs, and 146 participants (37.9%) relied primarily on informal sources. Only 164 professionals (42.6%) indicated that they routinely counseled patients about DFIs involving traditional foods or herbal products. Notably, only 73 participants (19.0%) had ever reported a DFI-related adverse event. These patterns suggest possible deficiencies in current curricular content, continuing professional education, and cultural alignment of counseling practices.

**Conclusion:**

The findings indicate potential gaps in healthcare professionals’ preparedness to manage DFIs effectively, particularly in relation to traditional food practices and pharmacovigilance. Tentatively, this may reflect shortcomings in both undergraduate curricula and post-graduate training programs. There may be value in integrating culturally sensitive DFI training into pharmacy and medical education, supported by continuing professional development initiatives. National guidelines tailored to local dietary contexts and patient beliefs could also enhance the quality of DFI-related counseling and reporting.

## Introduction

Understanding drug–food interactions (DFIs) is essential to optimizing therapeutic outcomes and ensuring medication safety. DFIs can alter drug absorption, metabolism, and overall efficacy, potentially leading to therapeutic failures or adverse reactions. Comprehensive knowledge of DFIs is required among healthcare professionals, particularly pharmacists, physicians, and nurses, to ensure effective management and prevention of such interactions. Practical strategies, such as adjusting the timing of drug administration relative to meals, have been shown to reduce the likelihood of clinically significant interactions (1, 2).

The prevalence and clinical consequences of DFIs are well documented. For example, dietary fiber-rich foods have been shown to interfere with the absorption of levothyroxine, resulting in sub-therapeutic responses and poor disease control (3). Similarly, variations in the intake of omega-3 fatty acids can modify the pharmacological effects of warfarin, highlighting the importance of dietary consistency in patients receiving anticoagulant therapy (4). Such examples demonstrate the practical importance of DFI awareness for both healthcare providers and patients.

Globally, the integration of drug–food interaction (DFI) education into healthcare curricula has been recognized as a crucial step toward improving medication safety and therapeutic outcomes. Systematic reviews and comparative studies have indicated that structured DFI education, tailored to pharmacists, physicians, and other healthcare professionals, enhances clinical decision-making and patient counseling skills (5–8). Despite this, international analyses have revealed considerable variation in the inclusion and depth of DFI-related content across medical, pharmacy, and nursing programs (9, 10). Evidence also shows that healthcare providers frequently underestimate the impact of dietary components on drug efficacy and safety, highlighting the need for educational reform and continuing professional development in this area (11–13). Collectively, these findings underscore the global call for standardized DFI modules and practical training integrated into undergraduate curricula and CPD programs to equip healthcare professionals with the knowledge and counseling competencies necessary for optimal patient care (14–16).

Despite growing evidence, awareness of DFIs among healthcare professionals remains inconsistent across practice settings. Studies suggest that many practitioners possess limited knowledge of the clinical significance of DFIs or the mechanisms through which food components influence drug bioavailability (2, 17). Educational interventions targeting these knowledge gaps have proven effective in enhancing DFI-related competencies and improving medication safety outcomes. Therefore, incorporating DFI education into undergraduate curricula and continuing professional development programs is crucial to ensuring that healthcare professionals are well prepared to counsel patients appropriately (18).

Knowledge retention and practical application may be strengthened through the adoption of innovative educational strategies in healthcare training programs. Problem-based learning and case-based discussions can help students and professionals apply DFI concepts to real-world clinical scenarios. Additionally, simulation-based training and interactive digital modules have demonstrated effectiveness in improving comprehension and decision-making related to medication–food interactions (19, 20). Such approaches not only improve knowledge but also foster critical thinking and communication skills essential for patient counseling.

As the understanding of pharmacotherapy continues to evolve, the need to educate healthcare professionals about DFIs becomes increasingly urgent. A well-informed workforce can significantly reduce preventable medication errors, enhance therapeutic efficacy, and contribute to safer patient care practices. Strengthening DFI education within professional curricula and clinical training frameworks represents a vital step toward improving public health outcomes and advancing medication safety (21).

Therefore, this study aimed to assess the knowledge, counseling practices, and educational gaps related to drug–food interactions among healthcare professionals in Saudi Arabia. Specifically, it sought to evaluate healthcare providers’ understanding of common DFIs, identify demographic and professional predictors associated with knowledge levels, and explore the need for enhanced educational strategies to strengthen safe medication practices within the Saudi healthcare context.

## Materials and methods

### Study design and setting

A cross-sectional study was conducted between October 2024 and January 2025 to assess the knowledge, attitudes, and practices (KAP) of healthcare professionals (HCPs) in Saudi Arabia regarding drug–food interactions (DFIs). The study employed a structured, self-administered online questionnaire distributed across five major geographic regions: North, South, East, West, and Central Saudi Arabia.

### Study population and sampling

The target population included licensed pharmacists, physicians, and nurses practicing in Saudi Arabia. Eligible participants were required to have at least 1 year of clinical experience and to provide informed consent prior to participation. Medical students, interns, unlicensed individuals, or those unwilling to participate were excluded.

Sample size calculation was performed using the Raosoft sample size calculator with a 95% confidence level, 5% margin of error, and an assumed response distribution of 50%, yielding a required sample of 385 respondents. A multistage stratified sampling technique was utilized. In the first stage, healthcare facilities were selected across the five geographic regions. In the second stage, participants were recruited using systematic random sampling from those facilities.

### Instrument development and validation

The survey instrument was developed following an extensive literature review and expert panel consultation. It consisted of six sections: demographic data, self-assessment of DFI familiarity, knowledge of DFIs, knowledge of medication timing, attitudes toward DFIs, and DFI-related practices.

Content validity was assessed by a panel of clinical pharmacy and pharmacology experts. A pilot test involving 20 healthcare professionals was conducted to assess clarity and feasibility; data from the pilot were excluded from the final analysis. Internal consistency was acceptable, with Cronbach’s alpha values of 0.78 for knowledge, 0.72 for attitudes, and 0.82 for practices.

### Data collection

The final survey was hosted on Microsoft Forms and distributed electronically via professional networks and social media platforms. Data were collected anonymously over a 3-month period (15 October 2024 to 15 January 2025). Participation was voluntary, and responses were de-identified prior to analysis.

### Scoring of outcomes

Knowledge of DFIs was assessed using nine true/false/don’t know items, with one point awarded per correct answer (range: 0–9). Knowledge of medication timing was assessed using seven items (range: 0–7). Attitude scores were calculated from four statements using a 3-point Likert scale (disagree = 1, unsure = 2, agree = 3), with a total score range of 4–12. Practice scores were based on five items rated on a 5-point Likert scale (never = 1 to always = 5), producing a total score range of 5–25.

### Statistical analysis

Data were analyzed using R software (version 4.4.2) and RStudio (version 2024.9.1.394). Descriptive statistics included medians and interquartile ranges (IQRs) for non-normally distributed continuous variables, and frequencies and percentages for categorical variables. Normality was assessed using the Shapiro–Wilk test.

Inferential analysis was conducted using the Wilcoxon rank-sum test (for dichotomous variables) and the Kruskal–Wallis test (for variables with > 2 categories). Variables with a *p*-value < 0.05 in bivariate analysis were included in multivariable linear regression models to identify independent predictors of knowledge, attitude, and practice scores. Regression coefficients (β), 95% confidence intervals (CIs), and *p*-values were reported. All tests were two-sided, with a significance threshold set at *p* < 0.05.

### Ethical considerations

The study protocol was reviewed and approved by the Biomedical Research Ethics Committee of Umm Al-Qura University, Makkah, Saudi Arabia (Approval No. HAPO-02-K-012-2025-01-2450). All methods were performed in accordance with the relevant guidelines and regulations, and the study was conducted in accordance with the principles of the Declaration of Helsinki. Written informed consent was obtained electronically from all participants prior to data submission. Data confidentiality and participant anonymity were maintained throughout the study.

## Results

### Demographic characteristics and self-perceptions on knowledge and practice of DFIs

A total of 385 participants were included in this cross-sectional study. Most respondents were aged between 30 and 39 years (46.8%), followed by those aged 22–29 years (37.1%). The sample consisted of 59.2% males and 40.8% females. Most participants were pharmacists (63.4%), followed by senior pharmacists (9.6%) and general practitioners (7.3%). The majority (88.6%) obtained their highest degree in Saudi Arabia. Regarding professional experience, 44.9% had more than 10 years of experience, while 29.1% had 1–5 years. The primary work settings were hospitals (40.9%) and pharmacies (40.4%). The southern region had the highest representation in the workplace (36.6%), followed by the western region (25.7%) and the central region (13.2%). Regarding familiarity with DFIs, 63.6% reported being somewhat familiar, and 31.2% indicated being very familiar. Most participants (68.3%) received formal training in DFIs during their undergraduate or graduate studies. DFIs were occasionally encountered by 54.8% of the respondents, and 64.9% reported being somewhat confident in identifying clinically significant DFIs (Table 1).

**TABLE 1:** Demographic characteristics and self-perceptions on knowledge and practice of drug-food interactions.

Characteristic	Description
**Age**	
22–29 years	143 (37.1%)
30–39 years	180 (46.8%)
40–49 years	58 (15.1%)
50–59 years	3 (0.8%)
Above 60 years	1 (0.3%)
**Gender**	
Male	228 (59.2%)
Female	157 (40.8%)
**Professional job**	
Medical consultant	6 (1.6%)
Medical specialist	5 (1.3%)
General practitioner	28 (7.3%)
Medical resident	11 (2.9%)
Consultant pharmacist	12 (3.1%)
Senior pharmacist	37 (9.6%)
Pharmacist	244 (63.4%)
Nurse	24 (6.2%)
Others	18 (4.7%)
**Country of the highest degree**	
From Saudi Arabia	341 (88.6%)
Outside of Saudi Arabia	44 (11.4%)
**Years of experience in the current profession**	
<1 year	42 (10.9%)
1–5 Years	112 (29.1%)
6–10 Years	58 (15.1%)
More than 10 years	173 (44.9%)
Primary place of work*	
Hospital	157 (40.9%)
Clinic	11 (2.9%)
Hospital pharmacy	155 (40.4%)
Community pharmacy	45 (11.7%)
Others	16 (4.2%)
**Region of the workplace**	
Northern region	49 (12.7%)
Southern region	141 (36.6%)
Eastern region	45 (11.7%)
Western region	99 (25.7%)
Central region	51 (13.2%)
**Familiarity with drug-food interactions**	
Very familiar	120 (31.2%)
Somewhat familiar	245 (63.6%)
Not familiar	20 (5.2%)
**Ever received formal training on drug-food interactions**	
No	93 (24.2%)
Yes, during undergraduate/graduate studies	263 (68.3%)
Yes, through professional development programs	29 (7.5%)
**Frequency of encountering drug-food interactions in practice**	
Never	25 (6.5%)
Rarely	104 (27.0%)
Occasionally	211 (54.8%)
Frequently	45 (11.7%)
**Confidence in identifying clinically significant drug-food interactions**	
Not confident	34 (8.8%)
Somewhat confident	250 (64.9%)
Very confident	101 (26.2%)

n (%); *The variable had one missing record.

### Description of the scores of different domains

Participants had a median score of 6.0 out of 9.0 (IQR = 4.0–8.0) for knowledge regarding DFIs. For knowledge of the best timing of drug intake, the median score was 5.0 out of 7.0 (IQR = 4.0–6.0). Details of the knowledge scores are summarized in Table 2. Attitudes toward DFIs had a median score of 10.0 out of 12.0 (IQR = 10.0–12.0). The median score for practices related to DFIs was 17.0 out of 25.0 (IQR = 14.0–20.0).

**TABLE 2:** Description of the scores of different domains.

Characteristic	Median (Q1–Q3)	Mean ± SD	Min-Max	N of items
Knowledge regarding drug-food interactions	6.0 (4.0–8.0)	5.6 ± 2.4	0.0–9.0	9
Knowledge regarding the best timing of drug intake	5.0 (4.0–6.0)	4.6 ± 1.6	0.0–7.0	7
Attitudes toward drug-food interactions	10.0 (10.0–12.0)	10.4 ± 1.4	5.0–12.0	4
Practice of drug-food interactions	17.0 (14.0–20.0)	16.7 ± 4.4	5.0–25.0	5

SD, standard deviation.

### Knowledge regarding DFIs

**FIGURE 1:**
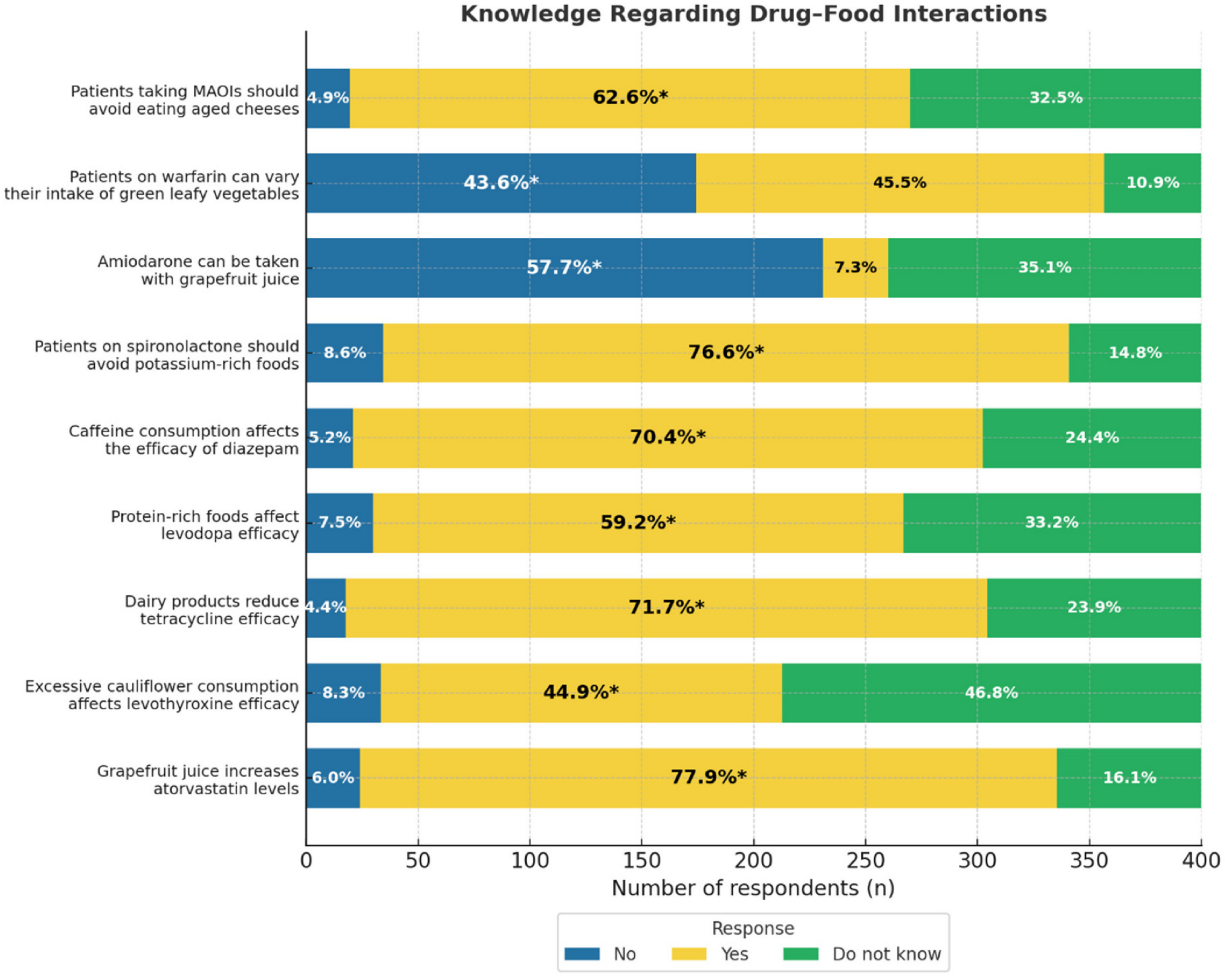
Knowledge regarding drug-food interactions bar graph. It represents the percentage of respondents’ awareness of drug-food interactions for MAOIs, warfarin, amiodarone, spironolactone, diazepam, levodopa, tetracycline, levothyroxine, and atorvastatin. Three response categories: No, Yes, Do not know. Percentages inside each bar indicate the proportion of respondents selecting each option, with an asterisk (*) marking the correct answer.

Several key DFIs were correctly identified by the majority of participants. Specifically, 77.9% correctly reported that grapefruit juice increased atorvastatin levels, and 76.6% recognized that patients on spironolactone should avoid potassium-rich foods. Furthermore, 71.7% were aware that dairy products can reduce the efficacy of tetracyclines, and 70.4% acknowledged that caffeine can affect the effectiveness of diazepam. Correct responses were also recorded for interactions involving protein-rich foods and levodopa (59.2%) and MAOIs with aged cheeses (62.6%). However, only 44.9% correctly identified that excessive consumption of cauliflower affected the efficacy of levothyroxine. Notably, 57.7% of the participants correctly stated that amiodarone should not be taken with grapefruit juice, and 43.6% correctly indicated that patients on warfarin should not freely vary their intake of green leafy vegetables (Figure 1).

### Factors and predictors of high scores of general knowledge regarding DFIs

Compared with medical consultants, nurses had significantly lower general knowledge scores (β = −2.09, 95% CI = −4.04 to −0.15, *p* = 0.036). Participants working in clinics scored significantly lower than those working in hospitals (β = −2.27, 95% CI = −3.63, −0.91, *p* = 0.001). Participants who reported being “somewhat familiar” (β = −0.94, 95% CI, −1.51 to −0.37, *p* = 0.001) or “not familiar” (β = −2.04, 95% CI, −3.26 to −0.82, *p* = 0.001) with DFIs had lower knowledge scores than those who were “very familiar.” Those who received DFI training during undergraduate or graduate studies had higher scores than those who did not (β = 0.68, 95% CI, 0.11–1.25, *p* = 0.019). Compared to participants who never encountered DFIs in practice, those who rarely (β = 1.18, 95% CI, 0.20–2.16, *p* = 0.019) and occasionally (β = 0.99, 95% CI, 0.03–1.96, *p* = 0.044) encountered them had significantly higher knowledge scores (Table 3).

**TABLE 3:** Factors and predictors of high general knowledge scores regarding drug-food interactions.

Variable	Inferential analysis		Multivariable regression		
	Median (IQR)	*P-*value	Beta	95% CI	*P-*value
Age		0.003			
22–29 years	7.0 (5.0, 8.0)		Reference	Reference	
30–39 years	6.0 (4.0, 8.0)		–0.45	–0.98, 0.08	0.096
40–49 years	5.0 (3.0, 7.0)		–0.30	–1.02, 0.43	0.424
50 years or more	5.0 (3.0, 7.5)		0.46	–1.63, 2.54	0.669
Gender		0.001			
Male	6.0 (3.0, 7.5)		Reference	Reference	
Female	6.0 (5.0, 8.0)		0.36	–0.12, 0.85	0.139
Professional job		< 0.001			
Medical consultant	6.0 (5.0, 8.0)		Reference	Reference	
Medical specialist	4.0 (4.0, 6.0)		–0.32	–2.83, 2.18	0.800
General practitioner	5.5 (2.5, 8.0)		–0.66	–2.60, 1.28	0.505
Medical resident	8.0 (2.0, 8.0)		–1.09	–3.23, 1.05	0.319
Consultant pharmacist	7.0 (6.0, 8.0)		0.32	–1.81, 2.45	0.770
Senior pharmacist	7.0 (6.0, 8.0)		0.65	–1.27, 2.57	0.506
Pharmacist	6.0 (4.0, 8.0)		–0.19	–2.03, 1.66	0.844
Nurse	3.0 (1.0, 5.0)		–2.09	–4.04, –0.15	0.036
Others	4.5 (4.0, 6.0)		–0.79	–2.84, 1.26	0.451
Country of the highest degree		0.934			
From Saudi Arabia	6.0 (4.0, 8.0)				
Outside of Saudi Arabia	6.5 (4.0, 7.5)				
Years of experience in the current profession		0.069			
<1 year	7.0 (6.0, 8.0)				
1–5 years	6.0 (4.0, 8.0)				
6–10 years	6.0 (4.0, 8.0)				
More than 10 years	6.0 (3.0, 8.0)				
Primary place of work		< 0.001			
Hospital	6.0 (4.0, 8.0)		Reference	Reference	
Clinic	2.0 (0.0, 5.0)		–2.27	–3.63, –0.91	0.001
Hospital pharmacy	6.0 (4.0, 8.0)		–0.19	–0.74, 0.36	0.498
Community pharmacy	7.0 (6.0, 8.0)		0.82	–0.01, 1.64	0.053
Others	5.5 (3.0, 6.0)		–1.06	–2.16, 0.04	0.061
Region of the workplace		0.002			
Northern region	6.0 (4.0, 8.0)		Reference	Reference	
Southern region	5.0 (3.0, 7.0)		–0.16	–0.86, 0.55	0.661
Eastern region	6.0 (4.0, 8.0)		0.04	–0.82, 0.90	0.929
Western region	6.0 (5.0, 8.0)		0.20	–0.54, 0.95	0.589
Central region	6.0 (4.0, 8.0)		–0.15	–0.98, 0.68	0.720
Familiarity with drug-food interactions		< 0.001			
Very familiar	8.0 (6.0, 8.0)		Reference	Reference	
Somewhat familiar	6.0 (4.0, 7.0)		–0.94	–1.51, –0.37	0.001
Not familiar	2.0 (1.0, 5.0)		–2.04	–3.26, –0.82	0.001
Ever received formal training on drug-food interactions		< 0.001			
No	5.0 (2.0, 7.0)		Reference	Reference	
Yes, during undergraduate/graduate studies	7.0 (5.0, 8.0)		0.68	0.11, 1.25	0.019
Yes, through professional development programs	5.0 (3.0, 7.0)		0.33	–0.62, 1.29	0.494
Frequency of encountering drug-food interactions in practice		< 0.001			
Never	4.0 (0.0, 6.0)		Reference	Reference	
Rarely	6.0 (4.0, 7.5)		1.18	0.20, 2.16	0.019
Occasionally	6.0 (4.0, 8.0)		0.99	0.03, 1.96	0.044
Frequently	7.0 (5.0, 8.0)		0.98	–0.14, 2.09	0.086
Confidence in identifying clinically significant drug-food interactions		< 0.001			
Not confident	5.0 (2.0, 6.0)		Reference	Reference	
Somewhat confident	6.0 (4.0, 7.0)		0.03	–0.82, 0.89	0.939
Very confident	8.0 (6.0, 8.0)		0.99	–0.02, 2.00	0.056

IQR, interquartile range. Kruskal-Wallis rank sum test; Wilcoxon rank sum test. CI, Confidence Interval.

### Knowledge regarding the best timing of drug intake

**FIGURE 2:**
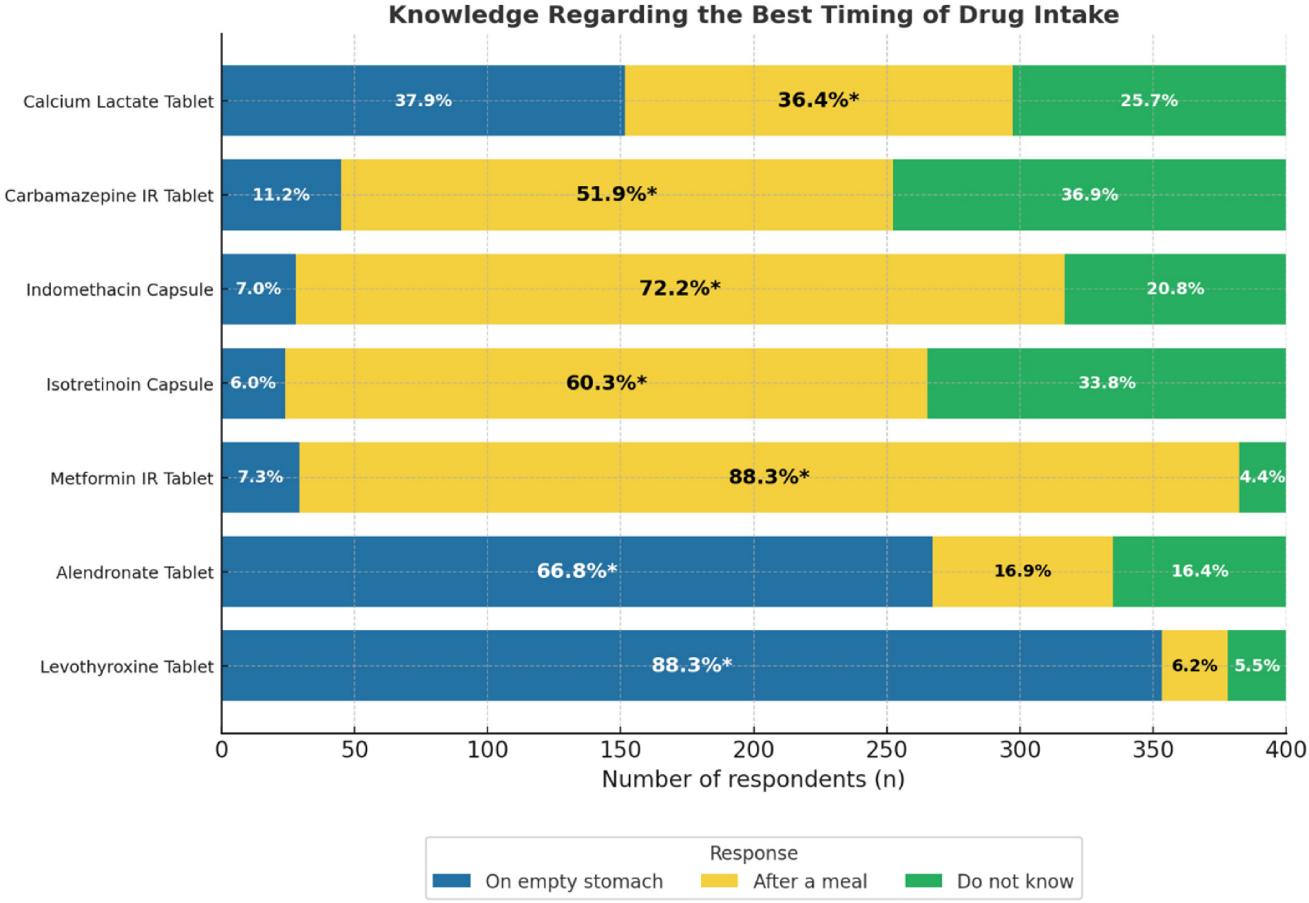
Knowledge regarding the best timing of drug bar graph. It is showing the responses for seven drugs: Calcium Lactate, Carbamazepine IR, Indomethacin, Isotretinoin, Metformin IR, Alendronate, and Levothyroxine Tablets. Responses are divided into three categories: “On empty stomach,” “After a meal,” and “Do not know.” Percentages inside each bar indicate the proportion of respondents selecting each option, with an asterisk (*) marking the correct answer.

Most participants demonstrated a good knowledge of the appropriate timing of drug administration. The correct timing was most frequently identified for levothyroxine tablets and metformin Immediate Release (IR) tablets, with 88.3% of respondents selecting “on an empty stomach” and “after a meal,” respectively. Similarly, 72.2% correctly indicated that indomethacin capsules should be taken after a meal, and 66.8% identified that the correct timing for alendronate tablets was on an empty stomach. The correct responses were lower for isotretinoin capsules (60.3%) and carbamazepine IR tablets (51.9%). For calcium lactate, 36.4% correctly selected “after a meal,” while 37.9% incorrectly selected “on an empty stomach” (Figure 2).

### Factors and predictors of high knowledge scores regarding the best timing of drug intake

Significantly higher knowledge scores regarding the best timing of drug intake were observed among female participants compared with males (β = 0.37, 95% CI, 0.03–0.71, *p* = 0.032). Compared to those working in hospitals, participants working in hospital pharmacies had slightly lower scores (β = −0.41, 95% CI, −0.82 to 0.00, *p* = 0.049). Additionally, participants who reported not being familiar with DFIs had significantly lower scores than those who were very familiar (β = −1.20, 95% CI, −2.12 to −0.28, *p* = 0.011) (Table 4).

**TABLE 4:** Factors and predictors of high knowledge scores regarding the best timing of drug intake.

Variable	Inferential analysis		Multivariable regression		
	Median (IQR)	*P-*value	Beta	95% CI	*P-*value
Age		0.371			
22–29 years	5.0 (3.0, 6.0)				
30–39 years	5.0 (4.0, 6.0)				
40–49 years	5.0 (3.0, 6.0)				
50 years or more	3.5 (3.0, 5.0)				
Gender		0.007			
Male	5.0 (3.0, 6.0)		Reference	Reference	
Female	5.0 (4.0, 6.0)		0.37	0.03, 0.71	0.032
Professional job		0.011			
Medical consultant	4.5 (4.0, 6.0)		Reference	Reference	
Medical specialist	4.0 (4.0, 6.0)		0.67	–1.22, 2.56	0.485
General practitioner	5.0 (3.0, 6.0)		–0.03	–1.49, 1.44	0.973
Medical resident	6.0 (4.0, 6.0)		0.37	–1.25, 1.98	0.658
Consultant pharmacist	5.5 (2.5, 6.0)		–0.12	–1.74, 1.50	0.883
Senior pharmacist	5.0 (4.0, 6.0)		0.82	–0.63, 2.27	0.269
Pharmacist	5.0 (4.0, 6.0)		0.69	–0.70, 2.07	0.332
Nurse	4.0 (2.0, 5.0)		–0.70	–2.16, 0.77	0.352
Others	3.0 (3.0, 5.0)		–0.12	–1.67, 1.42	0.874
Country of the highest degree		0.052			
From Saudi Arabia	5.0 (4.0, 6.0)				
Outside of Saudi Arabia	4.0 (3.0, 6.0)				
Years of experience in the current profession		0.139			
< 1 year	5.0 (3.0, 6.0)				
1–5 years	5.0 (3.0, 6.0)				
6–10 years	5.0 (4.0, 6.0)				
More than 10 years	5.0 (4.0, 6.0)				
Primary place of work		0.024			
Hospital	5.0 (4.0, 6.0)		Reference	Reference	
Clinic	4.0 (2.0, 5.0)		–0.80	–1.83, 0.22	0.126
Hospital pharmacy	5.0 (4.0, 6.0)		–0.41	–0.82, 0.00	0.049
Community pharmacy	5.0 (4.0, 6.0)		–0.04	–0.61, 0.53	0.893
Others	4.0 (3.0, 5.0)		–0.83	–1.66, 0.00	0.051
Region of the workplace		0.234			
Northern region	6.0 (4.0, 6.0)				
Southern region	4.0 (3.0, 6.0)				
Eastern region	5.0 (4.0, 6.0)				
Western region	5.0 (3.0, 6.0)				
Central region	5.0 (4.0, 6.0)				
Familiarity with drug-food interactions		< 0.001			
Very familiar	6.0 (5.0, 6.0)		Reference	Reference	
Somewhat familiar	4.0 (3.0, 6.0)		–0.31	–0.74, 0.11	0.149
Not familiar	3.0 (1.5, 5.0)		–1.20	–2.12, –0.28	0.011
Ever received formal training on drug-food interactions		0.015			
No	4.0 (3.0, 6.0)		Reference	Reference	
Yes, during undergraduate/graduate studies	5.0 (4.0, 6.0)		0.18	–0.24, 0.61	0.402
Yes, through professional development programs	4.0 (3.0, 6.0)		0.03	–0.69, 0.75	0.936
Frequency of encountering drug-food interactions in practice		< 0.001			
Never	4.0 (2.0, 5.0)		Reference	Reference	
Rarely	4.0 (3.0, 6.0)		0.44	–0.30, 1.18	0.248
Occasionally	5.0 (4.0, 6.0)		0.73	0.00, 1.46	0.052
Frequently	5.0 (4.0, 6.0)		0.39	–0.45, 1.24	0.360
Confidence in identifying clinically significant drug-food interactions		< 0.001			
Not confident	4.0 (3.0, 5.0)		Reference	Reference	
Somewhat confident	5.0 (3.0, 6.0)		–0.08	–0.72, 0.57	0.820
Very confident	6.0 (4.0, 6.0)		0.42	–0.34, 1.18	0.281

IQR, interquartile range; CI, Confidence Interval.Kruskal-Wallis rank sum test; Wilcoxon rank sum test.

### Attitudes toward DFIs

**FIGURE 3:**
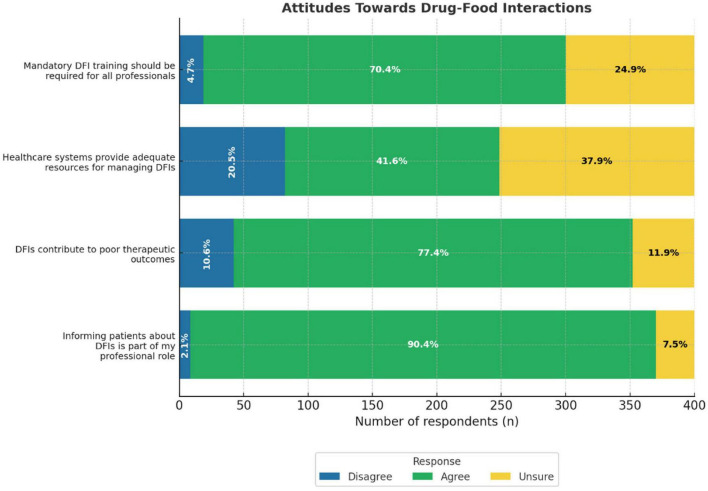
Attitudes towards drug-food interactions bar chart. It shows the responses from healthcare professionals who disagreed, were unsure, or agreed with each statement. The statments include mandatory DFI training, healthcare resources, DFI impact on outcomes, and informing patients.

The majority of participants demonstrated positive attitudes toward DFIs. Most notably, 90.4% agreed that informing patients about DFIs was part of their professional role. Similarly, 77.4% of patients agreed that DFIs contributed to poor therapeutic outcomes. Additionally, 70.4% of the participants supported the notion that mandatory training on DFIs is necessary for all HCPs. By contrast, only 41.6% agreed that the healthcare system provides adequate resources for managing DFIs, indicating a perceived gap in institutional support (Figure 3).

### Factors and predictors of high scores of attitudes toward DFIs

Female participants had significantly more positive attitudes toward DFIs than male participants (β = 0.35, 95% CI = 0.06–0.64, *p* = 0.017). Compared to those who were very familiar with DFIs, somewhat familiar participants had lower attitude scores (β = −0.50, 95% CI = −0.84 to −0.15, *p* = 0.005). Participants who rarely (beta = 0.88, 95% CI, 0.28–1.48, *p* = 0.004), occasionally (β = 1.34, 95% CI, 0.75–1.93, *p* < 0.001), or frequently (β = 1.04, 95% CI, 0.35–1.73, *p* = 0.003) encountered DFIs in practice had significantly higher attitude scores than those who never experienced them (Table 5).

**TABLE 5:** Factors and predictors of high scores of attitudes toward drug-food interactions.

Variable	Inferential analysis		Multivariable regression		
	Median (IQR)	*P-*value	Beta	95% CI	*P-*value
Age		0.005			
22–29 years	11.0 (10.0, 12.0)		Reference	Reference	
30–39 years	11.0 (10.0, 12.0)		0.08	–0.22, 0.38	0.609
40–49 years	10.0 (10.0, 10.0)		–0.41	–0.83, 0.01	0.055
50 years or more	10.0 (10.0, 11.0)		–0.10	–1.38, 1.19	0.884
Gender		< 0.001			
Male	10.0 (10.0, 11.0)		Reference	Reference	
Female	11.0 (10.0, 12.0)		0.35	0.06, 0.64	0.017
Professional job		0.372			
Medical consultant	11.0 (10.0, 12.0)				
Medical specialist	11.0 (10.0, 11.0)				
General practitioner	11.0 (10.5, 12.0)				
Medical resident	11.0 (10.0, 12.0)				
Consultant pharmacist	11.5 (10.0, 12.0)				
Senior pharmacist	11.0 (10.0, 11.0)				
Pharmacist	10.0 (10.0, 12.0)				
Nurse	10.0 (10.0, 11.0)				
Others	11.0 (9.0, 12.0)				
Country of the highest degree		0.573			
From Saudi Arabia	10.0 (10.0, 12.0)				
Outside of Saudi Arabia	10.0 (10.0, 11.0)				
Years of experience in the current profession		0.484			
<1 year	10.0 (9.0, 11.0)				
1–5 years	10.0 (10.0, 12.0)				
6–10 years	10.5 (10.0, 12.0)				
More than 10 years	10.0 (10.0, 12.0)				
Primary place of work		0.295			
Hospital	11.0 (10.0, 12.0)				
Clinic	10.0 (8.0, 11.0)				
Hospital pharmacy	10.0 (10.0, 12.0)				
Community pharmacy	11.0 (10.0, 12.0)				
	10.0 (8.0, 11.0)				
Region of the workplace		0.063			
Northern region	10.0 (10.0, 12.0)				
Southern region	10.0 (10.0, 12.0)				
Eastern region	11.0 (10.0, 12.0)				
Western region	11.0 (10.0, 11.0)				
Central region	10.0 (10.0, 12.0)				
Familiarity with drug-food interactions		< 0.001			
Very familiar	11.0 (10.0, 12.0)		Reference	Reference	
Somewhat familiar	10.0 (10.0, 11.0)		–0.50	–0.84, –0.15	0.005
Not familiar	9.0 (8.0, 12.0)		–0.71	–1.44, 0.02	0.059
Ever received formal training on drug-food interactions		0.007			
No	10.0 (8.0, 11.0)		Reference	Reference	
Yes, during undergraduate/graduate studies	11.0 (10.0, 12.0)		0.11	–0.24, 0.46	0.532
Yes, through professional development programs	11.0 (10.0, 12.0)		0.01	–0.55, 0.56	0.981
Frequency of encountering drug-food interactions in practice		< 0.001			
Never	9.0 (8.0, 10.0)		Reference	Reference	
Rarely	10.0 (9.0, 11.0)		0.88	0.28, 1.48	0.004
Occasionally	11.0 (10.0, 12.0)		1.34	0.75, 1.93	< 0.001
Frequently	10.0 (10.0, 12.0)		1.04	0.35, 1.73	0.003
Confidence in identifying clinically significant drug-food interactions		0.010			
Not confident	10.0 (8.0, 11.0)		Reference	Reference	
Somewhat confident	10.0 (10.0, 12.0)		0.01	–0.52, 0.54	0.981
Very confident	11.0 (10.0, 12.0)		–0.22	–0.85, 0.41	0.489

IQR, interquartile range; CI, Confidence Interval. Kruskal-Wallis rank sum test; Wilcoxon rank sum test.

### Practice of DFIs

**FIGURE 4:**
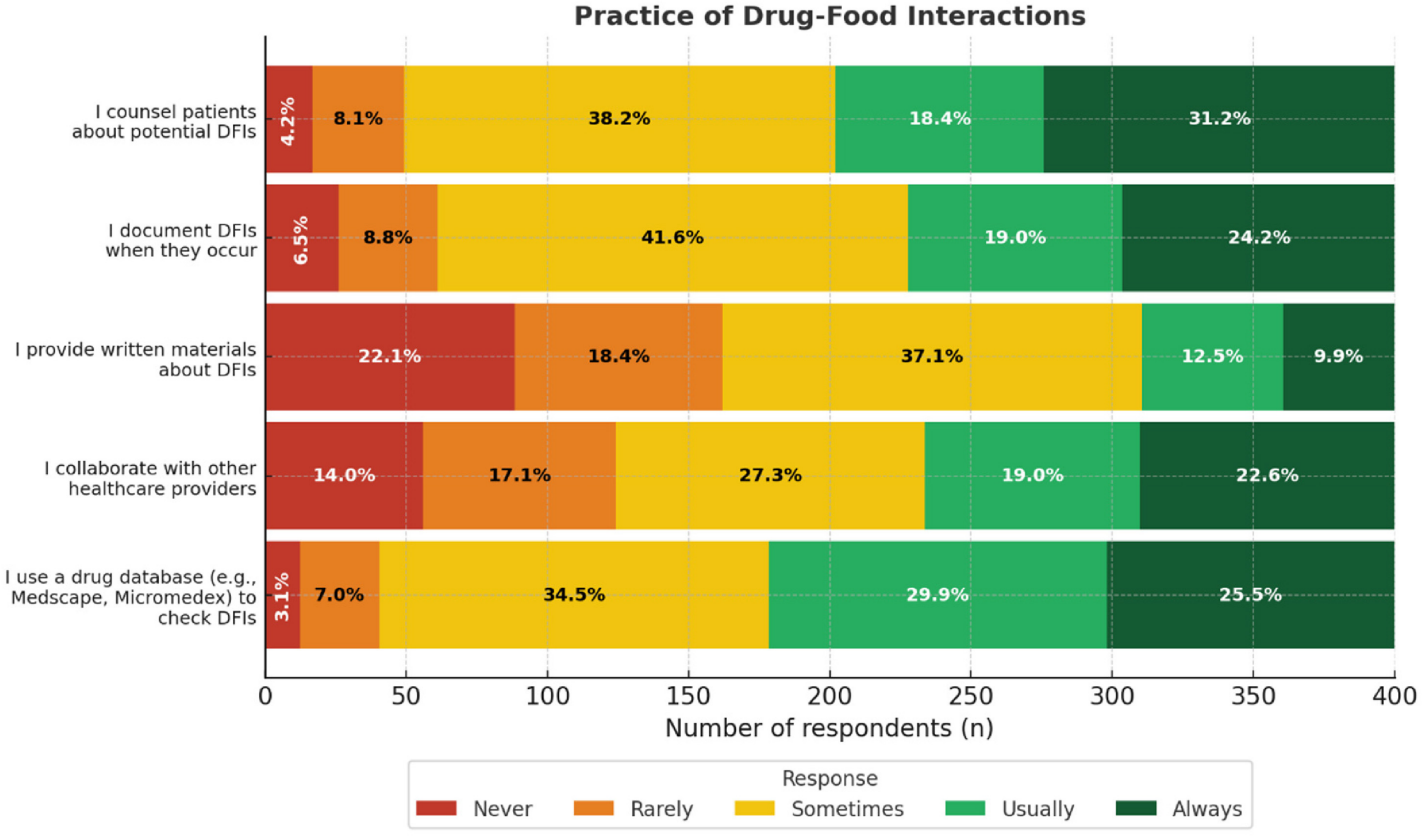
Practice of drug-food interactions encounters among healthcare providers bar chart. It shows the percentage of respondents who answered “Never,” “Rarely,” “Sometimes,” “Usually,” or “Always” for each practice Categories include using a drug database, collaborating with healthcare providers, providing written materials, documenting, and counseling patients about potential interactions.

A total of 55.4% of the participants reported that they usually or always counsel patients about potential DFIs. Similarly, 41.6% indicated that they usually or always documented DFIs when they occurred. Drug databases were commonly used to check for interactions, with 49.6% reporting frequent use. Collaboration with other HCPs on DFI management was reported by 43.2% of participants, while only 22.4% reported that they usually or always provided written materials about DFIs to patients (Figure 4).

### Factors and predictors of high scores of practices of DFIs

Compared to medical consultants, higher practice scores were observed among medical specialists (β = 5.09, 95% CI, 0.21–9.96, *p* = 0.042), general practitioners (β = 4.27, 95% CI, 0.46–8.08, *p* = 0.029), medical residents (β = 5.43, 95% CI, 1.20–9.66, *p* = 0.012), consultant pharmacists (β = 6.96, 95% CI, 2.73–11.2, *p* = 0.001), senior pharmacists (β = 5.40, 95% CI, 1.65–9.16, *p* = 0.005), pharmacists (β = 7.35, 95% CI, 3.77–10.9, *p* < 0.001), nurses (β = 5.48, 95% CI, 1.61–9.35, *p* = 0.006), and those classified as “others” (β = 5.59, 95% CI, 1.56–9.62, *p* = 0.007). Participants who were somewhat familiar with DFIs had lower scores than those who were very familiar (β = −2.33, 95% CI, −3.45 to −1.22, *p* < 0.001). Compared to those who never encountered DFIs, higher practice scores were reported by participants who rarely (β = 2.51, 95% CI, 0.58–4.44, *p* = 0.011), occasionally (β = 2.73, 95% CI, 0.83– 4.62, *p* = 0.005), and frequently (β = 3.09, 95% CI, 0.89–5.30, *p* = 0.006) encountered them. Those who reported being somewhat confident (β = 2.12, 95% CI, 0.42–3.82, *p* = 0.015) or very confident (β = 2.71, 95% CI, 0.73–4.68, *p* = 0.008) in identifying clinically significant DFIs also had significantly higher practice scores than those who were not confident (Table 6).

**TABLE 6:** Factors and predictors of high scores of practice of drug-food interactions.

Variable	Inferential analysis		Multivariable regression		
	Median (IQR)	*P-*value	Beta	95% CI	*P-*value
Age		0.605			
22–29 years	17.0 (14.0, 20.0)					
30–39 years	17.0 (14.0, 20.0)					
40–49 years	16.0 (13.0, 19.0)					
50 years or more	14.5 (13.5, 18.5)					
Gender		0.811			
Male	17.0 (14.0, 20.0)				
Female	17.0 (14.0, 19.0)				
Professional job		< 0.001			
Medical consultant	11.5 (10.0, 12.0)		Reference	Reference	
Medical specialist	11.0 (10.0, 22.0)		5.09	0.21, 9.96	0.042
General practitioner	15.0 (13.0, 18.0)		4.27	0.46, 8.08	0.029
Medical resident	15.0 (14.0, 19.0)		5.43	1.20, 9.66	0.012
Consultant pharmacist	17.0 (16.0, 22.0)		6.96	2.73, 11.2	0.001
Senior pharmacist	17.0 (14.0, 20.0)		5.40	1.65, 9.16	0.005
Pharmacist	17.0 (15.0, 20.0)		7.35	3.77, 10.9	< 0.001
Nurse	14.5 (12.5, 17.0)		5.48	1.61, 9.35	0.006
Others	15.5 (13.0, 17.0)		5.59	1.56, 9.62	0.007
Country of the highest degree		0.452			
From Saudi Arabia	16.0 (14.0, 20.0)				
Outside of Saudi Arabia	17.0 (14.5, 20.0)				
Years of experience in the current profession		0.961			
< 1 Year	17.0 (13.0, 20.0)				
1–5 Years	16.5 (14.0, 19.5)				
6–10 Years	16.0 (14.0, 20.0)				
More than 10 years	16.0 (14.0, 20.0)				
Primary place of work		0.168			
Hospital	16.0 (13.0, 19.0)				
Clinic	17.0 (14.0, 22.0)				
Hospital pharmacy	17.0 (15.0, 19.0)				
Community pharmacy	17.0 (15.0, 20.0)				
Others	14.0 (9.5, 19.5)				
Region of the workplace		0.109			
Northern region	17.0 (15.0, 19.0)				
Southern region	16.0 (13.0, 20.0)				
Eastern region	18.0 (15.0, 21.0)				
Western region	17.0 (14.0, 20.0)				
Central region	15.0 (13.0, 18.0)				
Familiarity with drug-food interactions		< 0.001			
Very familiar	19.0 (16.0, 22.0)		Reference	Reference	
Somewhat familiar	15.0 (13.0, 19.0)		–2.33	–3.45, –1.22	< 0.001
Not familiar	15.5 (10.0, 20.0)		–1.04	–3.41, 1.32	0.388
Ever received formal training on drug-food interactions		0.010			
No	16.0 (12.0, 18.0)		Reference	Reference	
Yes, during undergraduate/graduate studies	17.0 (15.0, 20.0)		0.42	–0.68, 1.53	0.454
Yes, through professional development programs	16.0 (12.0, 19.0)		0.87	–1.02, 2.75	0.367
Frequency of encountering drug-food interactions in practice		< 0.001			
Never	13.0 (10.0, 16.0)		Reference	Reference	
Rarely	16.0 (12.0, 20.0)		2.51	0.58, 4.44	0.011
Occasionally	17.0 (15.0, 19.0)		2.73	0.83, 4.62	0.005
Frequently	18.0 (15.0, 21.0)		3.09	0.89, 5.30	0.006
Confidence in identifying clinically significant drug-food interactions		< 0.001			
Not confident	14.5 (9.0, 17.0)		Reference	Reference	
Somewhat confident	16.0 (14.0, 20.0)		2.12	0.42, 3.82	0.015
Very confident	18.0 (15.0, 21.0)		2.71	0.73, 4.68	0.008

IQR: interquartile range; CI, Confidence Interval. Kruskal-Wallis rank sum test; Wilcoxon rank sum test.

## Discussion

Substantial deficiencies in healthcare professionals’ knowledge and practices concerning drug–food interactions were revealed (DFIs), particularly within the context of pharmacy practice in Saudi Arabia. Among the 385 participants, 231 (60.0%) demonstrated limited awareness of DFI mechanisms. This indicates a broader shortfall in education and training, possibly due to limited curricular coverage of DFIs and insufficient continuing professional development opportunities. Comparable deficits have been reported regionally, as community pharmacists in Palestine struggled to identify common DFIs despite recognizing their clinical relevance, and similar limitations were observed among healthcare workers in Ethiopia (22, 23). Collectively, these patterns reflect a systemic educational challenge across the Middle East and Africa that warrants comprehensive reform.

The lack of structured education emerges as a plausible explanation for these knowledge gaps. In this study, 198 participants (51.4%) reported never receiving formal instruction on DFIs, while 146 (37.9%) indicated that their knowledge was primarily acquired informally. These results align with international evidence highlighting insufficient curricular integration of DFI topics within pharmacy education (24, 25). Similar concerns have been raised in Malaysia and Poland, where inadequate DFI instruction contributes to knowledge gaps and medication errors (26, 27). The importance of embedding DFI content into pharmacy curricula to enhance awareness and practical counseling skills is underscored by these educational deficiencies.

Counseling practices also appear to be influenced by educational background. Only 164 respondents (42.6%) reported routinely discussing DFIs related to traditional foods or herbal remedies with patients. This pattern mirrors international findings showing that, while pharmacists generally recognize their responsibility to counsel patients, many lack sufficient confidence or depth of knowledge to provide culturally sensitive advice (28, 29). Moreover, reliance on unverified online resources for DFI information—reported in other community pharmacy studies—raises concerns about the accuracy and safety of patient counseling (30, 31). These findings highlight the need for structured, evidence-based counseling frameworks that integrate both clinical and cultural knowledge.

Underreporting of DFI-related adverse events represents another critical issue. In this study, only 73 participants (19.0%) had ever submitted a pharmacovigilance report. This aligns with previous research documenting low levels of reporting among Saudi healthcare professionals, often linked to limited awareness of reporting systems and uncertainty regarding procedures (32, 33). Regionally, Alshammari et al. and Garashi et al. noted that underdeveloped pharmacovigilance infrastructure and training deficiencies remain major barriers, while Alzubiedi et al. reported that the COVID-19 pandemic further disrupted reporting processes (34–36). Addressing these gaps through education, standardized reporting protocols, and integration of DFI-specific examples in training programs could improve the safety surveillance system and reporting culture.

Building on these findings, pharmacy curricula in Saudi Arabia may not adequately address the complexities of DFIs. The high proportion of participants lacking formal DFI instruction reinforces the need for curricular reform. Existing programs across the MENA region prioritize biomedical and regulatory content while neglecting applied patient-facing competencies such as counseling and communication (25, 26). Simulation-based learning, cultural case studies, and integration of traditional medicine examples into pharmacotherapy teaching could bridge this gap and prepare students for real-world counseling.

Internationally, pharmacy education models in high-income countries have successfully incorporated interprofessional learning and cultural competence modules (27, 37). Adapting these frameworks to the Saudi context could enhance patient-centered education while respecting local dietary and cultural practices. Cultural alignment is particularly crucial, as pharmacists working in regions with prevalent traditional herbal use must tailor counseling to patients’ dietary customs to improve adherence and trust (38, 39). Regional calls for culturally contextualized DFI counseling guidelines further support this approach (40).

Continuing professional development (CPD) offers a practical mechanism to address knowledge retention and practice gaps post-graduation. CPD participation has been shown to enhance pharmacist competence and confidence in areas such as dietary counseling and adverse event reporting (41, 42). Effective CPD should be grounded in local dietary habits and patient communication norms to ensure relevance and sustainability. Research suggests that structured CPD initiatives directly improve professional performance, particularly when aligned with competency-based standards (43, 44). Integrating mandatory, DFI-specific modules into existing CPD frameworks could therefore enhance the ability of pharmacists to manage complex medication regimens safely.

Cultural competence and interprofessional collaboration represent vital dimensions of safe medication practice. Studies emphasize that pharmacists’ understanding of patients’ cultural and dietary patterns significantly affects counseling quality and patient adherence (45–47). Collaborative learning models, including interprofessional education (IPE), foster shared responsibility among healthcare providers, reducing the likelihood of DFI-related errors (22). Integrating cultural competence and interprofessional training within pharmacy curricula ensures that healthcare professionals can navigate complex interactions involving traditional diets, supplements, and prescribed medications effectively (38, 39).

While these findings provide valuable insights, several limitations should be acknowledged. The cross-sectional design limits causal inference, and self-reported data may be subject to recall and social desirability bias, especially concerning counseling behaviors. The convenience sampling approach constrains generalizability, and the study did not assess participants’ personal cultural beliefs or traditional medicine practices, which may influence DFI counseling performance. Additionally, although participants were recruited from all major Saudi regions using a stratified approach, regional representation was uneven, with a higher proportion of respondents from the Southern region (36.6%). This imbalance may reflect differential response rates and could introduce non-response bias, meaning that the findings may be more reflective of the Southern region’s healthcare professionals than of the entire national population. Future studies should consider proportional stratification or weighting to achieve a more balanced representation across regions.

Collectively, the results suggest that pharmacy education and professional training in Saudi Arabia would benefit from explicit instruction on DFI identification, management, and culturally competent counseling. Integrating case-based and simulation learning, pharmacovigilance modules, and patient communication exercises can strengthen applied knowledge and skills. Expanding CPD programs to include DFI-specific content and incorporating culturally relevant examples would further reinforce pharmacist readiness. At the policy level, developing national DFI counseling guidelines and reporting protocols under the Saudi Commission for Health Specialties (SCFHS) could standardize practice and align with Vision 2030’s commitment to healthcare excellence. Future research should adopt mixed-methods designs to explore how traditional beliefs and cultural factors influence DFI counseling, ultimately informing evidence-based educational interventions and safer pharmacotherapy practices.

## Conclusion

This study identified significant gaps in healthcare professionals’ knowledge and counseling practices regarding drug–food interactions (DFIs) in Saudi Arabia. The findings highlight the need for structured educational reform and continuous professional development focusing on DFI management, pharmacovigilance, and culturally competent counseling. Applied knowledge and patient communication may be strengthened through the integration of simulation-based learning, pharmacovigilance training, and case-based instruction into pharmacy curricula, together with tailored CPD programs. Strengthening healthcare provider competency in these areas will ultimately enhance medication safety, patient adherence, and overall therapeutic outcomes.

## Data Availability

The raw data supporting the conclusions of this article will be made available by the authors, without undue reservation.
